# Colonization With Antibiotic-Resistant Bacteria in a Hospital and Associated Communities in Guatemala: An Antibiotic Resistance in Communities and Hospitals (ARCH) Study

**DOI:** 10.1093/cid/ciad222

**Published:** 2023-07-05

**Authors:** Brooke M Ramay, Carmen Castillo, Laura Grajeda, Lucas F Santos, Juan Carlos Romero, Maria Renee Lopez, Andrea Gomez, Mark Caudell, Rachel M Smith, Ashley Styczynski, Carolyn T A Herzig, Susan Bollinger, Mariangeli Freitas Ning, Jennifer Horton, Sylvia Omulo, Guy H Palmer, Celia Cordon-Rosales, Douglas R Call

**Affiliations:** Center for Health Studies, Universidad del Valle de Guatemala, Guatemala City, Guatemala Department, Republic of Guatemala; Paul G. Allen School for Global Health, Washington State University, Pullman, Washington, USA; Center for Health Studies, Universidad del Valle de Guatemala, Guatemala City, Guatemala Department, Republic of Guatemala; Center for Health Studies, Universidad del Valle de Guatemala, Guatemala City, Guatemala Department, Republic of Guatemala; Center for Health Studies, Universidad del Valle de Guatemala, Guatemala City, Guatemala Department, Republic of Guatemala; Center for Health Studies, Universidad del Valle de Guatemala, Guatemala City, Guatemala Department, Republic of Guatemala; Center for Health Studies, Universidad del Valle de Guatemala, Guatemala City, Guatemala Department, Republic of Guatemala; Center for Health Studies, Universidad del Valle de Guatemala, Guatemala City, Guatemala Department, Republic of Guatemala; Paul G. Allen School for Global Health, Washington State University, Pullman, Washington, USA; Division of Healthcare Quality Promotion, Centers for Disease Control and Prevention, Atlanta, Georgia, USA; Division of Healthcare Quality Promotion, Centers for Disease Control and Prevention, Atlanta, Georgia, USA; Division of Healthcare Quality Promotion, Centers for Disease Control and Prevention, Atlanta, Georgia, USA; Division of Healthcare Quality Promotion, Centers for Disease Control and Prevention, Atlanta, Georgia, USA; Central America Regional Office, Centers for Disease Control and Prevention, Guatemala City, Guatemala Department, Republic of Guatemala; Paul G. Allen School for Global Health, Washington State University, Pullman, Washington, USA; Paul G. Allen School for Global Health, Washington State University, Pullman, Washington, USA; Washington State University Global Health–Kenya, Nairobi, Nairobi County, Kenya; Paul G. Allen School for Global Health, Washington State University, Pullman, Washington, USA; Center for Health Studies, Universidad del Valle de Guatemala, Guatemala City, Guatemala Department, Republic of Guatemala; Paul G. Allen School for Global Health, Washington State University, Pullman, Washington, USA; Paul G. Allen School for Global Health, Washington State University, Pullman, Washington, USA

**Keywords:** antimicrobial resistance, public health, communities, hospitals, Guatemala

## Abstract

**Background:**

We estimated the prevalence of colonization with extended-spectrum cephalosporin-resistant Enterobacterales (ESCrE) and carbapenem-resistant Enterobacterales (CRE) from a hospital and associated communities in western Guatemala.

**Methods:**

Randomly selected infants, children, and adults (<1, 1–17, and ≥18 years, respectively) were enrolled from the hospital (n = 641) during the coronavirus disease 2019 (COVID-19) pandemic, March to September 2021. Community participants were enrolled using a 3-stage cluster design between November 2019 and March 2020 (phase 1, n = 381) and between July 2020 and May 2021 (phase 2, with COVID-19 pandemic restrictions, n = 538). Stool samples were streaked onto selective chromogenic agar, and a Vitek 2 instrument was used to verify ESCrE or CRE classification. Prevalence estimates were weighted to account for sampling design.

**Results:**

The prevalence of colonization with ESCrE and CRE was higher among hospital patients compared to community participants (ESCrE: 67% vs 46%, *P* < .01; CRE: 37% vs 1%, *P* < .01). Hospital ESCrE colonization was higher for adults (72%) compared with children (65%) and infants (60%) (*P* < .05). Colonization was higher for adults (50%) than children (40%) in the community (*P* < .05). There was no difference in ESCrE colonization between phase 1 and 2 (45% and 47%, respectively, *P* > .05), although reported use of antibiotics among households declined (23% and 7%, respectively, *P* < .001).

**Conclusions:**

While hospitals remain foci for ESCrE and CRE colonization, consistent with the need for infection control programs, community prevalence of ESCrE in this study was high, potentially adding to colonization pressure and transmission in healthcare settings. Better understanding of transmission dynamics and age-related factors is needed.

## Teaser

Colonization with extended-spectrum cephalosporin (ESCrE) and carbapenem-resistant Enterobacterales was significantly higher for hospitalized patients compared with household participants. Community ESCrE prevalence was unchanged despite a 3-fold reduction in reported use of antibiotics during a period of COVID-19 pandemic restrictions.

Bacterial infections from antimicrobial-resistant pathogens occur globally. The antimicrobial resistance burden is relatively well understood in higher-income countries, but data are limited in low- and middle-income countries where surveillance is often sporadic [1, 2]. Surveillance networks, including the Global Antimicrobial Resistance and Use Surveillance System (GLASS), estimate the prevalence of antimicrobial resistance from clinical isolates with a standardized surveillance methodology. The Americas, however, have had the lowest GLASS participation with no Central American countries enrolled as of 2020 [3]. Of the 35 countries in the Americas, 12 have published national action plans in the past 7 years, of which 2 are Central American but do not include Guatemala [4]. The Pan American Health Organization Latin American Network for Antimicrobial Resistance Surveillance (ReLAVRA) reports resistance of clinical isolates that are collected from 19 Latin American countries on its regional dashboard (https://www3.paho.org/data/index.php/en/mnu-topics/antimicrobial-resistance/571-amr-vig-en.html). Data from Guatemala indicate a 6-fold increase in the prevalence of carbapenem-resistant *Klebsiella pneumoniae* among clinical isolates between 2011 and 2019 (6% to 36%) [5]. Additionally, a systematic review of published literature estimated that third-generation cephalosporin-resistant Enterobacterales comprise 25%–58% of clinical isolates in Latin America [6]. Nevertheless, inconsistent reporting methods and dependence on clinical isolate data prevent unbiased estimation of prevalence at a population level; therefore, trend analysis is fraught with challenges.

One approach to overcome this bias problem is to estimate the prevalence of antimicrobial-resistant colonizing bacteria. Existing literature on colonization focuses on hospitalized patients [7] and risk of subsequent infection among sick patients, which reflects a patient-level focus rather than a population or public health perspective [8–10]. When colonization studies are conducted at the population level, the results can describe the prevalence of antimicrobial resistance throughout a community and the interplay between communities and hospitals while also providing a platform to evaluate prevention programs and interventions [11]. When carefully designed, such studies generate unbiased estimates of colonization among target populations and may inform ways to limit transmission pathways [12]. With this motivating rationale, we conducted a study to estimate the prevalence of colonization with antimicrobial-resistant bacteria in the western highlands of Guatemala, with a focus on hospitalized patients and healthy community members from the hospital catchment area.

## MATERIALS AND METHODS

This study was part of the Antibiotic Resistance in Communities and Hospitals (ARCH) studies conducted across 6 countries to estimate the prevalence of colonization with extended-spectrum cephalosporin-resistant Enterobacterales (ESCrE) and carbapenem-resistant Enterobacterales (CRE) in healthcare and associated community settings [11]. Our study deviated from the published protocol by (1) enrolling children of all ages in the hospital and community settings instead of limiting sampling to adults and (2) by not reporting data on the prevalence of methicillin-resistant *Staphylococcus aureus*.

### Study Sites

We enrolled participants from the western highlands of Guatemala (department of Quetzaltenango) where both Spanish-speaking mestizo and Mam- and Quiche-speaking indigenous populations reside in urban and rural settings. Urban locations were defined per Guatemalan census guidelines as cities, towns, villages, or neighborhoods with >2000 inhabitants, provided that 51% or more of households have electricity and piped water [13]. Rural locations included all areas not meeting the urban definition. Urban and rural residents are served by a single tertiary referral hospital, Hospital Regional San Juan de Dios de Occidente in Quetzaltenango (253 adult beds, 49 pediatric beds, and 118 infant beds), which was used for the hospital component of the study. The community component was executed within the catchment area of the hospital, defined by data previously collected via an integrated, health facility–based surveillance system for respiratory, diarrheal, and febrile illness [14].

### Hospital Sampling

Hospital wards (excluding outpatient services) were visited repeatedly to enroll eligible participants. Individuals of all ages were randomly selected from a ward-level census on the day of enrollment if admitted by 8:00 Am of the sampling date and if not already recruited for the study. Patients were excluded if they had documented severe neutropenia (absolute neutrophil count <1700 cells/µL for people ≤12 years old or <1000 cells/µL for people >12 years old) or if they had gastrointestinal bleeding or known COVID-19.

### Community Sampling

Enrollment of community participants (adults aged ≥18 years, children aged 1–17 years, and infants aged <1 year) was divided into 3 stages. We first partitioned the study area into clusters (polygons on a map) where each cluster included an estimated 75 residents based on WorldPop reference data [15]. Mapped clusters were generated using GridSample [15, 16]. Clusters were then randomly selected, and satellite images (Google Maps) were used to manually count the number of household rooftops within each polygon. The presence of households was confirmed on site, noting the GPS location of households, the name of the head of household, and the number of household members (Supplementary Figure 1). Fifteen households were randomly selected for enrollment from each cluster (if ≤15 households were present, all were approached). Consent was obtained from the head of the selected household, and consent (or consent and assent for children aged 7–17 years) was obtained from 1 randomly selected household member. Individuals were eligible for inclusion if they had lived in the household for at least 6 months and excluded if they presented with fever, diarrhea, or cough at the time of interview and/or specimen collection. Households or individuals undergoing quarantine or isolation due to COVID-19 exposure or infection were also excluded. We initiated the community study in November 2019 (phase 1) but stopped on 20 March 2020 due to COVID-19 pandemic restrictions. We completed the community component between July and May of 2021 (phase 2) when some COVID-19 public health restrictions were still in place. Between November 2020 and May 2021, children <15 years old were enrolled exclusively to increase child representation in the sample.

### Data Collection

Community questionnaires included self-reported syndromic illnesses (febrile illness, diarrhea, pneumonia, influenza-like illness) and antibiotics taken in the 30 days prior to enrollment. Hospital data collection included a questionnaire and information from medical records. Stool samples were collected within 24 hours of hospital enrollment and within 3 days of community enrollment. The community and hospital study protocols were approved by the Universidad del Valle de Guatemala–Center for Health Studies Research Ethics committee (194-04-2019 and 202-10-2019, respectively) and by the Guatemalan Ministry of Health Ethics Committee (16-2019 and 49-2019, respectively).

### Laboratory Methods

Stool samples were collected in stool cups and transported at 4°C to a local laboratory. FLOQSwabs were used to prepare stool swab samples that were then placed in eSwab liquid Amies medium plus 1-mL sterile 40% glycerol (in 1 × phosphate-buffered saline) before storage at −80°C. Thawed samples were streaked onto MacConkey agar (Hardy Diagnostics) as a positive control for gram-negative bacteria, and onto CHROMagar extended-spectrum β-lactamase and CHROMagar mSuperCarba agar plates followed by overnight incubation at 37°C. If samples did not yield gram-negative bacteria on MacConkey agar plates, they were subjected to enrichment by culture in MacConkey broth. Up to 3 morphologically distinct colonies were recovered from each chromogenic plate (up to 6 colonies total) and were stored at −80°C for later characterization. If no colonies were present after enrichment and plating on MacConkey media, the sample was classified as “no growth” and was not included in prevalence estimates. All no-growth samples yielded gram-positive cultures when incubated with tryptic soy broth.

A bioMérieux VITEK 2 Compact was used to identify and classify antibiotic susceptibility for each isolate using VITEK 2 GN ID and AST-GN84 test cards, respectively. Breakpoints for antibiotic susceptibility classifications followed the Clinical and Laboratory Standards Institute M100-S31 [17] (Supplementary Table 1). Isolates identified as Enterobacterales were considered ESCrE if they were resistant to ceftriaxone and susceptible or intermediate to all carbapenem antibiotics tested. Enterobacterales isolates were classified as CRE if they exhibited resistance to ≥1 of the tested carbapenems.

### Statistical Methods

For the hospital study, we weighted prevalence estimates according to the number of patients in the ward, stratified by age category. Overall estimates of weighted prevalence were compared by age, sex, and ethnicity using χ^2^ tests. Estimates for the community study were weighted by the product of the cluster selection weight, cluster response weight, household selection weight, household response weight, individual selection weight, and individual response weight (Supplementary Table 2). Individual response weights for participants enrolled from November to May 2021 considered the number of children <15 years old in the household exclusively. Census data for the catchment area (2018) were used to normalize weighted prevalence estimates according to distributions of age, sex, and urban/rural households within the catchment area [13]. Resulting prevalence estimates were compared using χ^2^ tests. Before pooling ESCrE colonization data across phase 1 and phase 2, we verified that there were no significant differences between prevalence estimates for these 2 phases for sex, age, ethnicity, and general location (Supplementary Table 3). Multidrug resistance was characterized according to the difficult-to-treat resistance (DTR) phenotype, which includes nonsusceptibility to all β-lactams, including carbapenems, and fluoroquinolones against which the organism was tested [18].

## RESULTS

In total, 965 community participants and 979 hospital participants were enrolled, of which 919 (95%) and 641 (65%), respectively, provided stool samples (Supplementary Figure 2). The proportion of adults in the community study (48%; median, 41 years [range, 18–93 years]) was similar for hospital participants (45%; median, 42 years [range, 18–86 years]). The median age of hospitalized children (aged 1–17 years) was 4 years, and for hospitalized infants between 1 and 11 months of age, the median age was 3 months. The median age of children (1–17 years) in the community was 6 years. Thirty-three infants (<1 year old) were enrolled as community participants and these data were pooled with the children age group. Compared to hospital participants, more community participants were female (68% vs 49%) and of indigenous ethnicity (61% vs 44%). Sixty-seven percent of community participants were from rural households.

### Hospital Prevalence

**Figure 1.**
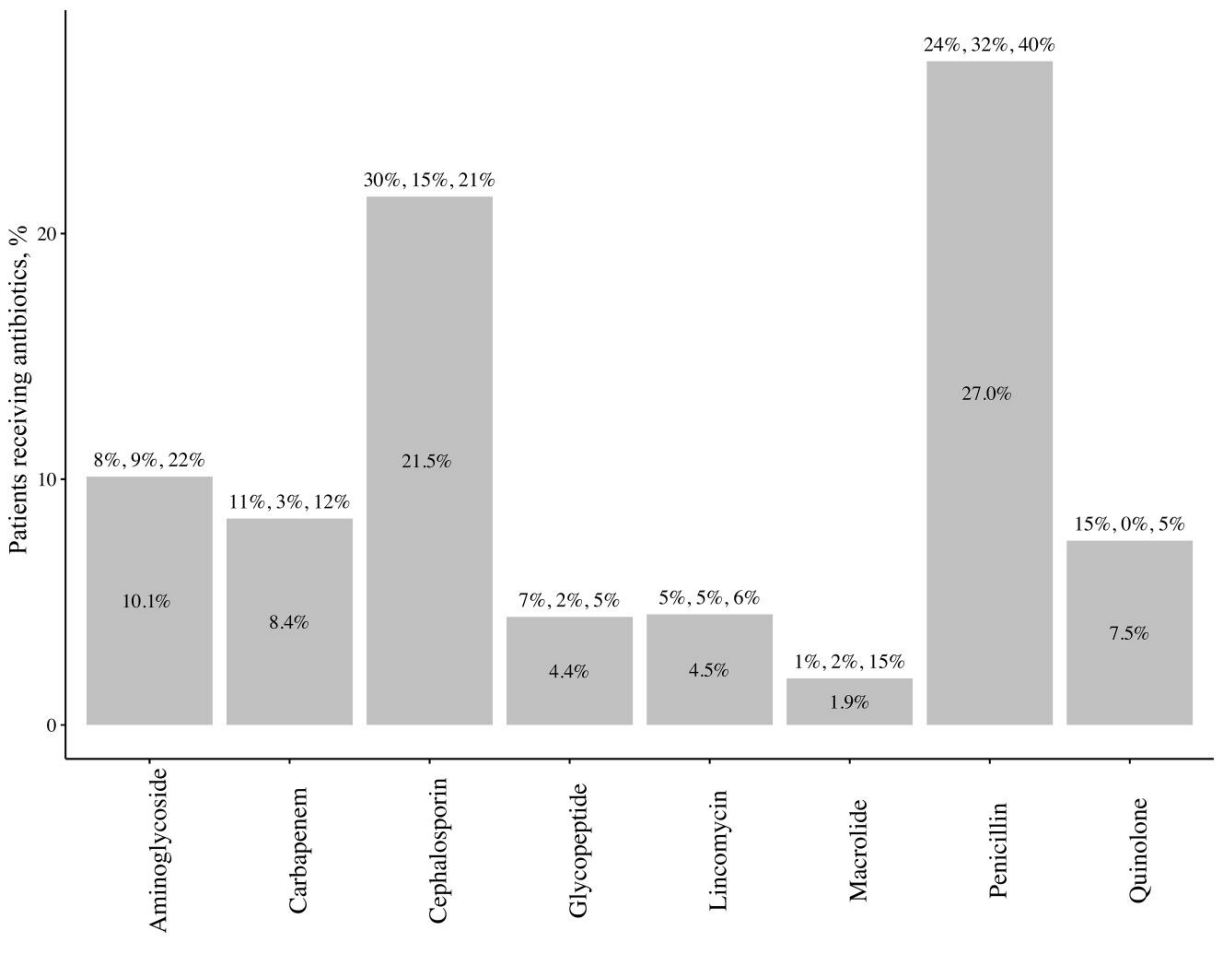
Percentage of hospitalized patients receiving antibiotics. Percentage for all ages (n = 641) is shown inside bars, and percentage by age (adults, n = 288; children, n = 155; infants, n = 198; respectively) is shown above bars. Data were abstracted from patient medical records and antibiotic classes used <5% for all age groups were excluded from the figure (fosfomycin, glycylcycline, polymyxin, sulfonamide, and tetracycline). *Klebsiella* are intrinsically resistant to ampicillin and ticarcillin. Some β-lactam antibiotics (eg, penicillin G and oxacillin) and other classes (glycopeptides, lincomycins, and macrolides) do not typically affect gram-negative bacteria such as *Escherichia coli*.

From the original 641 stool samples, 121 (19%) did not produce gram-negative bacteria after enrichment, and these were excluded from consideration in prevalence estimates. Most hospital participants were colonized with ESCrE (67%) with significant differences by age (72% in adults, 65% in children, and 60% in infants) (Table 1). The overall prevalence of CRE colonization was 37% with age differences that were not statistically different. Unweighted estimates are available (Supplementary Table 4). Among hospital patients, 16% met the DTR phenotypic classification (Supplementary Table 5). The 2 most used antibiotic classes were penicillins and cephalosporins (29.9% and 23.8%, respectively) (Figure 1). Notably, among the 121 patient samples that yielded no gram-negative bacteria, 60 (50%) were collected from infants between zero and 3 days old. The remaining participants (n = 61) received an average of 2.5 antibiotics during the time from admission to enrollment compared to an average of 2.0 for participants whose samples yielded gram-negative bacteria (*P* < .01).

**Table 1.** Weighted Prevalence Estimates for Extended-Spectrum Cephalosporin-Resistant Enterobacterales and Carbapenem-Resistant Enterobacterales Colonization of Hospital Participants for Which Gram-negative Bacteria Were Recovered on MacConkey Agar With or Without Enrichment

Characteristic	Population, No.	Weighted Prevalence^a^, ESCrE Positive (95% CI)	Weighted Prevalence, CRE Positive (95% CI)
Pooled across groups	641	67% (62%–72%)	37% (32%–42%)
Age group			
Adults (≥18 y)	288	**72%** (65%–78%)	40% (32%–47%)
Children (1–17 y)	155	**65%** (56%–75%)	29% (19%–38%)
Infants (<1 y)	198	**60%** (51%–68%)	37% (28%–46%)
Sex			
Female	313	69% (62%–75%)	34% (27%–41%)
Male	328	65% (58%–72%)	41% (33%–48%)
Ethnicity			
Indigenous	275	69% (62%–76%)	41% (33%–49%)
Mestizo	366	65% (59%–72%)	35% (28%–41%)

Samples were collected between March and September 2021.

Bold denotes *P* < .05; averages were compared using Pearson χ^2^ test, with a Rao and Scott adjustment.

Abbreviations: CI, confidence interval; CRE, carbapenem-resistant Enterobacterales; ESCrE, extended-spectrum cephalosporin-resistant Enterobacterales.

Weighted prevalence accounts for ward-level patient population by age class at the time of sampling. The estimated design effect for ESCrE colonization was 2.2 and the estimated design effect for CRE colonization was 2.4.

### Community Prevalence

All but 11 (1 adult and 10 children) stool samples provided by community participants yielded gram-negative bacteria when plated onto MacConkey agar plates without antibiotics. From 919 samples that yielded bacteria, on average, 1% of household participants were colonized with CRE isolates and 46% of household participants were positive for ESCrE colonization (Table 2). Unweighted prevalence estimates are available (Supplementary Table 6). Prevalence of ESCrE colonization was higher among adults (50%) compared with children (40%) (Table 2). Among community participants, 1% of isolates collected from stool samples met the DTR phenotypic classification (Supplementary Table 5).

**Table 2.** Weighted Average Prevalence Estimates for Extended-Spectrum Cephalosporin-Resistant Enterobacterales and Carbapenem-Resistant Enterobacterales Colonization of Community Participants (n = 919)

Group Included	Population, No.	Weighted Prevalence^a^, ESCrE Positive (CI)	Weighted Prevalence^a^, CRE Positive (CI)
Pooled across groups	919	46% (41%–51%)	1% (0%–2%)
Phase 1	381	45% (37%–53%)	…
Phase 2	538	47% (39%–54%)	…
Adults^b^	452	**50%** (44%–56%)	…
Children^b^	467	**40%** (33%–48%)	…
Female	625	49% (44%–54%)	…
Male	294	42% (35%–50%)	…
Indigenous	563	48% (41%–54%)	…
Mestizo	356	44% (36%–52%)	…
Urban	306	44% (40%–59%)	…
Rural	613	44% (38%–50%)	…

Samples were collected between November 2019 and March 2020 (phase 1) and between July 2020 and May 2021 (phase 2).

Bold denotes *P* < .03; averages were compared using Pearson χ^2^ test, with a Rao and Scott adjustment.

Abbreviations: CI, confidence interval; CRE, carbapenem-resistant Enterobacterales; ESCrE, extended-spectrum cephalosporin-resistant Enterobacterales.

Weighted prevalence accounts for geographic cluster, cluster response rate, household selection weight, household response weight, individual selection weight, and individual response weight followed by normalization based on census data for age, sex, and urban/rural location (Supplementary Table 2). The estimated design effect for ESCrE colonization was 2.28 and the estimated design effect for CRE colonization was 1.4. CRE isolates were sufficiently rare that their distribution was not compared by phase or demographic variable (…).

Adults were ≥18 years old and children were 1–17 years old. Three infants (<1 year old) were enrolled in the community study.

### Isolate Diversity and Resistance Phenotypes

There were 615 Vitek-confirmed community ESCrE isolates with unique antimicrobial resistance patterns of which 92.6% were *Escherichia coli*, and 10 CRE isolates of which 80% were *E. coli* (Supplementary Table 7). Hospital samples yielded 614 ESCrE isolates of which 66.9% were *E. coli* and 27.9% were *K. pneumoniae*. Hospital CRE isolates (n = 334) were similarly dominated by *E. coli* (45.8%) and *K. pneumoniae* (41.0%). Most hospital CRE were resistant to all tested β-lactams, with the most common additional resistance phenotypes including ciprofloxacin, levofloxacin, tetracycline, and sulfamethoxazole-trimethoprim (Supplementary Figures 3 and 4). ESCrE *E. coli* from hospital samples exhibited consistently higher proportions of resistance to other antibiotics compared with community isolates, with the highest proportions including resistance to ciprofloxacin, levofloxacin, sulfamethoxazole-trimethoprim, and tetracycline (Supplementary Figures 5–7).

### Phase 1 and 2 Syndromic Illness and Antibiotic Use

We performed a comparison of syndromic illness from phase 1 and phase 2 community participants for whom bacteria were recovered on MacConkey agar without antibiotics (n = 919). Reported syndromic illness during the 30 days prior to interview was significantly lower during phase 2 than during phase 1. Additionally, reported use of antibiotics at the household level was 3 times lower during phase 2 compared with phase 1 (7% vs 23%, respectively; Table 3). Changes in reported syndromes were not statistically different for adults and children (Supplementary Table 8).

**Table 3.** **Weighted Prevalence** ^a^ **of Reported Syndromic Illness in the Previous 30 Days for Community Participants (n = 919) During Phase 1 (Prepandemic) and Phase 2 (During COVID-19 Pandemic)**

Self-Reported Response	Phase 1	Phase 2	*P* Value
Influenza-like illness	12%	2%	<.001
Febrile illness	9%	4%	.008
Diarrhea	16%	5%	<.001
Pneumonia	4%	1%	<.001
Antibiotic consumption	23%	7%	<.001

Weighted prevalence accounts for geographic cluster, cluster response rate, household selection weight, household response weight, individual selection weight, and individual response weight followed by poststratification based on census data for age, sex, and urban/rural location. Weighted averages were normalized (Supplementary Table 2), and χ^2^ tests were used to compare between sample periods (phases 1 and 2).

## DISCUSSION

We found a high prevalence of ESCrE colonization among both hospital (67%) and community participants (46%). And although CRE prevalence in the community was rare (1%), the prevalence was concerningly high among hospitalized participants, including among infants, of whom 37% were colonized with CREs. This high CRE prevalence is consistent with potential for increased risk of CRE infections in hospitalized patients [19]. For example, surveillance of clinical isolates from Guatemala found that 36% exhibited carbapenem resistance [5], and the proportion of isolates classified as difficult-to-treat from this study (Supplementary Table 5) was also much greater for hospital (22%) than community (1%) participants. Consequently, there is potential risk that a high prevalence of colonization with these organisms translates into risk of multidrug-resistant infections.

The pattern of higher prevalence of ESCrE and CRE in hospital patients is consistent with findings from ARCH studies in Kenya [20] and Botswana [21]. Unlike these studies, however, there was an age difference, with adults from Guatemala having a higher prevalence of ESCrE compared with younger patients. This is consistent with a 1.4- to 2-fold higher frequency of cephalosporin use in adults (mostly ceftriaxone) (Figure 1). And although the difference between CRE colonization by age was not statistically significant (Table 1), the relative prevalence for adults, infants and children (40%, 37%, and 29%, respectively) was similar to the relative frequency of carbapenem use (11%, 12%, and 3%, respectively). These findings are consistent with the likelihood of colonization being higher with antibiotic use, either via direct selection or via greater opportunity for colonization with antibiotic-induced dysbiosis [22], particularly if colonization is occurring in the hospital itself. It is also possible that a priori colonized individuals are more likely to receive carbapenem and cephalosporin antibiotics. Importantly, because CRE were rarely encountered in the community, there may be limited dissemination of these strains into the community from the hospital.

Community ESCrE colonization was age distributed (adults 50%, children 40%), although reported use of antibiotics was not (15% and 14%, respectively; Supplementary Table 8). It is possible that the prevalence of ESCrE colonization in the community is attributable to the relatively high use of antibiotics reported by participants among phase 1 participants (23%; Table 3), although reported use was lower for phase 2 participants (7%). Earlier work in this community [23, 24] showed that both amoxicillin (without clavulanic acid) and tetracycline are easily purchased without a prescription. Amoxicillin can selectively favor ESCrE strains via allogeneous selection [25] whereas tetracycline might favor ESCrE strains via cross-selection (80% of ESCrE *E. coli* were resistant to tetracycline; Supplementary Figure 5).

Notably, use of antibiotics was significantly lower during phase 2 (6%), but prevalence of ESCrE colonization did not decrease (Tables 2 and 4). Assuming that self-reported use of antibiotics was not biased during phase 2, then the failure to detect a decline in ESCrE colonization may be due to an insufficient washout period (approximately 1 year) or may indicate that self-reported community use of antibiotics is not a dominant driver of ESCrE colonization [26]. It is also possible that the rebounding economic activity taking place as pandemic restrictions were lifted during phase 2 collection [27] altered risk factors for colonization in a way not captured in this study.

Some stool samples from hospital (19%) and community (1.2%) participants did not yield gram-negative bacteria even after enrichment. About half of these no-growth samples came from 0- to 3-day-old infants at the hospital, possibly due to underdeveloped gut microbiomes [28]. The remaining no-growth population at the hospital received a higher average number of antibiotics compared to stool samples from patients that yielded growth on MacConkey plates, although more analysis is needed to identify specific antibiotic risk practices that may contribute to no-growth outcomes. It is also possible that sample handling contributed to these differences, but our procedures were identical to the community study and were similar to a parallel project in Kenya [20], neither of which encountered a significant number of no-growth samples. Future studies of colonization with antimicrobial-resistant bacteria may wish to exclude 0- to 3-day-old patients to limit potential confounding from antibiotic exposure and microbiome maturity.

In conclusion, the prevalence of CRE and ESCrE was very high for hospital participants (37% and 67%, respectively), and community colonization with ESCrE was also high (46%). Moreover, hospital isolates exhibited a higher proportion of resistance to non-β-lactam antibiotics compared with community isolates, including a higher proportion of isolates that exhibited DTR phenotypes. If the higher prevalence of colonization in hospitals is nosocomial in origin, more effective infection control programs are needed [29]. And while antibiotic selection in the hospital likely plays an important role, the dramatic decrease in antibiotic use during the pandemic phase of community sampling without a commensurate reduction in ESCrE prevalence suggests that factors besides antibiotic use may underlie the high community prevalence of ESCrE.

We caution that the prevalence estimates provided by this study consider a complex set of survey weights to represent the Quetzaltenango regional hospital and community catchment area and, consequently, our estimates may not be applicable to the country as a whole. Antibiotic administration data from patient charts are a reliable representation of antibiotic exposure, but recall bias can introduce errors for the community study. Nevertheless, the apparent close correlation between reported antibiotic use and reported syndromic illness between the phase 1 and phase 2 sampling periods suggests that self-reported antibiotic use data were robust for the current study.

## Supplementary Data

Supplementary materials are available at *Clinical Infectious Diseases* online. Consisting of data provided by the authors to benefit the reader, the posted materials are not copyedited and are the sole responsibility of the authors, so questions or comments should be addressed to the corresponding author.

## Supplementary Material

ciad222_Supplementary_Data

## Notes

***Acknowledgments.*** The authors thank L. Godoy, A. Real, N. Lopez, V. Sicajau, and J. Guillermo Rivera for their assistance with data collection and sample processing. Staff and leadership from the Ministerio de Salud Pública y Asistencia Social, the Hospital Regional San Juan de Dios de Occidente, the Universidad del Valle de Guatemala, and the Centers for Disease Control and Prevention (CDC; Atlanta, Georgia, and the Central American Regional Office, Guatemala City) provided invaluable assistance for this work.

***Financial support.*** This work was supported by the Centers for Disease Control and Prevention (cooperative agreement U01 GH002241); the Wellcome Trust (award number 219832/Z/19/Z); and the Paul G. Allen School for Global Health at Washington State University.

***Supplement sponsorship.*** This article appears as part of the supplement “The Evolving Challenges of Antibiotic Resistance in Low- and Middle-Income Countries: Priorities and Solutions,” sponsored by the U.S. Centers for Disease Control and Prevention, and Health Security Partners.
