# Supplementary material for: Colonization With Antibiotic-Resistant Bacteria in a Hospital and Associated Communities in Guatemala: An Antibiotic Resistance in Communities and Hospitals (ARCH) Study
Source: Clin Infect Dis. 2023 Jul 5;77(Suppl 1):S82–8. doi: 10.1093/cid/ciad222 (PMC10321699; doi:10.1093/cid/ciad222)
Supplement: ciad222_Supplementary_Data [file ciad222_supplementary_data.docx]

**SUPPLEMENTAL MATERIALS**

**Colonization with antibiotic-resistant bacteria in a hospital and associated communities in Guatemala: An Antibiotic Resistance in Communities and Hospitals (ARCH) Study**

Brooke M. Ramay^1,2^, Carmen Castillo^1^, Laura Grajeda^1^, Lucas F. Santos^1^, Juan Carlos Romero^1^, Maria Renee Lopez^1^, Andrea Gomez^1^, Mark Caudell^2^, Rachel Smith^3^, Ashley Styczynski^3^, Carolyn Herzig^3^, Susan Bollinger^3^, Mariangeli Freitas Ning^4^, Jennifer Horton^2^, Sylvia Omulo^2,5^, Guy H. Palmer^2^, Celia Cordon-Rosales^1,2,^*, and Douglas R. Call^2,^*,‡

^1^Center for Health Studies, Universidad del Valle de Guatemala, Guatemala City, ^2^Paul G. Allen School for Global Health, Washington State University, Pullman, WA, USA, ^3^Division of Healthcare Quality Promotion, U.S. Centers for Disease Control and Prevention, Atlanta, USA, ^4^Central America Region, U.S. Centers for Disease Control and Prevention, Guatemala City, Guatemala, ^5^Washington State University Global Health-Kenya, Nairobi, Kenya

| **Supplementary Table 1.** Enterobacterales breakpoints (µg/ml) for Vitek2® antimicrobial susceptibility assays used for this study (CLSI M100-S31^a^). | | | | |
| --- | --- | --- | --- | --- |
|  | Abbreviation | Susceptible | Intermediate | Resistant |
| Amoxicillin + clavulanic acid | Amc | ≤ 8 | 16 | ≥ 32 |
| Ampicillin | Amp | ≤ 8 | 16 | ≥ 32 |
| Aztreonam | Atm | ≤ 4 | 8 | ≥ 16 |
| Cefazolin | Cz | ≤ 2 | 4 | ≥ 8 |
| Cefepime | Fep | ≤ 2 | 4-8 | ≥ 16 |
| Ceftriaxone | Cro | ≤ 1 | 2 | ≥ 4 |
| Ciprofloxacin | Cip | ≤ 0.25 | 0.5 | ≥ 1 |
| Ertapenem | Etp | ≤ 0.5 | 1 | ≥ 2 |
| Gentamicin | Gen | ≤ 4 | 8 | ≥ 16 |
| Imipenem | Imi | ≤ 1 | 2 | ≥ 4 |
| Levofloxacin | Lev | ≤ 0.5 | 1 | ≥ 2 |
| Meropenem | Mer | ≤ 1 | 2 | ≥ 4 |
| Nitrofurantoin | Nit | ≤ 32 | 64 | ≥ 128 |
| Piperacillin + tazobactam | Tzp | ≤ 16 | 32 - 64 | ≥128 |
| Trimethoprim + sulfamethoxazole | Sxt | ≤ 2/38 | -- | ≥ 4/76 |
| Tetracycline | Tet | ≤ 4 | 8 | ≥ 16 |
| ^a^CLSI. Performance standards for antimicrobial susceptibility testing. 31^st^ ed. CLSI supplement M100. Clinical and Laboratory Standards Institute; 2021. | | | | |

| **Supplementary Table 2.** The *svydesign* command from the R package *survey* (ver. 4.1-1) was used to calculate prevalence while accounting for design effects from the three-stage sampling frame that was used for the community study. Weights were assigned as the product of: ClusterSelectionWeight × ClusterResponseWeight × HouseholdSelectionWeight × HouseResponseWeight × IndividualSelectionWeight × IndividualResponseWeight. Variable definitions are provided below. | | |
| --- | --- | --- |
| **Selection weights** | **Quotient** | **Definition** |
| ClusterSelectionWeight | gk / gik / nk | gk = estimated total population in stratum k (the total population in the catchment area)  gik = estimated population in Primary Sampling Units (PSU) i in stratum k  nk = number of selected PSUs in stratum k |
| HouseholdSelectionWeight | mik / mik | mik = number of total households enumerated in PSU i and stratum k  mik = number of households selected in PSU i and stratum k |
| IndividualSelectionWeight | uijk / uijkx | uijk = number of total individuals enumerated in household j, PSU i and stratum k  uijkx = number of selected individuals enumerated in household j, PSU i and stratum k |
| **Response weights** |  |  |
| ClusterResponseWeight | nk / nkx | nk = number of selected PSUs in stratum k  nkx = number of found and sampled PSUs in stratum k |
| HouseResponseWeight | mik / mikx | mik = number of selected households in PSU i and stratum k  mikx = number of households that were identified and that responded in PSU i and stratum k |
| IndividualResponseWeight | uijk / uijkx | uijk = number of selected persons in household j, PSU i and stratum k  uijkx = number of individuals identified and that responded in household j, PSU i and stratum k |

| **Supplementary** **Table 3.** Normalized weight average prevalence (95% confidence interval) of ESCrE colonization for different demographic groups during phase 1 (pre-pandemic, n = 381) and phase 2 (COVID-19 pandemic, n = 538) of the community study. See Table S2 for additional details about weighting. Averages were compared using Pearson`s χ^2^, with a Rao and Scott adjustment (R package survey, ver. 4.1-1). | | | | |
| --- | --- | --- | --- | --- |
|  | **Phase 1** | **Phase 2** | **Difference** | ***P*** |
| Female | 49% (39-57%) | 50% (42-57%) | -1% | 0.83 |
| Male | 39% (28-52%) | 44% (32-56%) | -5% | 0.63 |
| Indigenous | 47% (37-56%) | 48% (40-55%) | -1% | 0.88 |
| Mestizo | 37% (23-51%) | 47% (39-55%) | -10% | 0.28 |
| Urban | 50% (37-63%) | 47% (39-55%) | 3% | 0.75 |
| Rural | 43% (34-52%) | 44% (36-51%) | -1% | 0.88 |
| Adult | 42% (35-49%) | 54% (43-64%) | -12% | 0.14 |
| Child | 44% (32-58%) | 41% (31-51%) | 0% | 0.68 |
|  | | | | |

| **Supplementary** **Table 4.** Unweighted prevalence estimates for ESCrE and CRE in hospital participants (n=641). Significant differences (χ^2^ test) in prevalence of ESCrE across age groups, shown in bold (*P*=0.05); all other comparisons were non-significant. | | | | |
| --- | --- | --- | --- | --- |
|  |  |  | Unweighted prevalence | |
|  |  | Population, n | ESCrE | CRE |
| Age groups | Adults | 288 | **70%** | 38% |
|  | Children | 155 | **66%** | 23% |
|  | Infants | 198 | **64%** | 31% |
|  |  |  |  |  |
| Sex | Female | 313 | 67% | 30% |
|  | Male | 328 | 66% | 34% |
|  |  |  |  |  |
| Ethnicity | Indigenous | 275 | 67% | 33% |
|  | Mestizo | 366 | 67% | 31% |

| **Supplementary** **Table 5.** Participants with at least one bacterial isolate from stool that would be classified as “Difficult to Treat” (DTR) had it been responsible for an infection. This table includes modified definitions and phenotypic combinations for the interested reader. | | |
| --- | --- | --- |
|  | **Community**  **(n = 919)**  **n (%)** | **Hospital**  **(n = 641)**  **N (%)** |
|  |  |  |
| DTR^a^ | 8 (1) | 140 (22) |
| Modified DTR^b^ | 11 (1) | 196 (22) |
| DTR + aminoglycoside resistance | 1 (0) | 89 (14) |
| DTR + tetracycline resistance | 5 (1) | 110 (17) |
| DTR + trimethoprim-sulfonamide^c^ resistance | 5 (1) | 125 (20) |
| DTR + resistance to two or more additional classes | 5 (1) | 126 (20) |
|  |  |  |
| ^a^Intermediate or resistant to all of the following: amoxicillin/clav, amoxicillin, aztreonam, cefazolin, cefepime, ceftriaxone, ciprofloxacin, ertapenem, imipenem, levofloxacin, meropenem, piperacillin/tazobactam  ^b^Modified DTR (DTR without carbapenems), intermediate or resistant to all of the following: amoxicillin/clav, amoxicillin, aztreonam, cefazolin, cefepime, ceftriaxone, ciprofloxacin, levofloxacin, piperacillin/tazobactam  ^c^Trimethoprim-sulfonamide refers to testing both drugs in combination (i.e., Sxt, Table S1). | | |

| **Supplementary** **Table 6.** Unweighted prevalence estimates for ESCrE in community participants (n = 919). Significant differences in prevalence were evident when comparing adults and children (*P* < 0.05). | | | |
| --- | --- | --- | --- |
|  | | Population, n | Unweighted prevalence |
|  |  |  |  |
| Phase | Pre-pandemic | 381 | 46% |
|  | During pandemic | 538 | 48% |
|  |  |  |  |
| Age group | Adults | 452 | 51% |
|  | Children | 467 | 44% |
|  |  |  |  |
| Sex | Female | 625 | 48% |
|  | Male | 294 | 45% |
|  |  |  |  |
| Ethnicity | Indigenous | 563 | 47% |
|  | Mestizo | 356 | 47% |
|  |  |  |  |
| Household type | Urban | 306 | 47% |
|  | Rural | 613 | 47% |

| **Supplementary** **Table 7.** Species or group identification for ESCrE and CRE isolates from community and hospital participants. Identity was determined using Vitek2® Gram-negative ID cards. This summary includes species from Enterobacterales that had unique antimicrobial resistance profiles from each stool sample. | | | | |
| --- | --- | --- | --- | --- |
| Species or group^a^ | Community, n (%) | | Hospital, n (%) | |
|  | ESCrE | CRE | ESCrE | CRE |
|  |  |  |  |  |
| All isolates | 615 | 10 | 614 | 334 |
|  |  |  |  |  |
| *Citrobacter amalonaticus* |  |  |  | 5 (1.5) |
| *Citrobater farmeri* |  |  |  | 2 (0.6) |
| *Citrobacter freundii* | 1 (0.2) |  | 3 (0.5) | 14 (4.2) |
| *Enterobacter aerogenes* | 1 (0.2) |  | 7 (1.1) | 1 (0.3) |
| *Enterobater asburiae* |  |  | 1 (0.2) |  |
| *Enterobacter cloacae* complex | 10 (1.6) | 1 (10.0) | 15 (2.4) | 16 (4.8) |
| *Escherichia coli* | 568 (92.4) | 8 (80.0) | 407 (66.3) | 153 (45.8) |
| *Escherichia coli* serovar O157:H7 | 2 (0.3) |  | 4 (0.7) | 1 (0.3) |
| *Escherichia fergusonii* |  |  | 1 (0.2) |  |
| *Klebsiella oxytoca* | 2 (0.3) |  | 25 (4.1) | 3 (0.9) |
| *Klebsiella pneumoniae* | 27 (4.4) | 1 (10.0) | 146 (23.8) | 134 (40.1) |
| *Pluralibacter gergoviae* |  |  |  | 1 (0.3) |
| *Proteus mirabilis* |  |  |  | 1 (0.3) |
| *Raoultella ornithinolytica* |  |  |  | 1 (0.3) |
| *Raoultella planticola* | 1 (0.2) |  | 2 (0.3) | 1 (0.3) |
| *Salmonella* group |  |  | 1 (0.2) |  |
| *Serratia ficaria* |  |  |  | 1 (0.3) |
| *Serratia odorifera* |  |  | 1 (0.2) |  |
| *Shigella sonnei* | 3 (0.5) |  |  |  |
|  |  |  |  |  |
| ^a^For the purposes of analysis, bacteria from the same genus were pooled. | | | | |

| **Supplementary** **Table 8.** Average reported syndromic illness in the previous 30 days for community participants (n = 919) by adults and children^a^. Data was pooled for phase I and phase II collection periods. | | | |
| --- | --- | --- | --- |
|  |  |  |  |
|  | Adult (n=452) | Children (n=467) | *P* |
|  |  |  |  |
| Influenza-like illness | 9% | 5% | 0.17 |
| Fever syndrome | 7% | 5% | 0.3 |
| Diarrhea | 12% | 7% | 0.1 |
| Pneumonia | 3% | 2% | 0.52 |
| Antibiotic consumption | 15% | 14% | 0.9 |
|  |  |  |  |
| ^a^Weighted prevalence accounts for geographic cluster, cluster response rate, household selection weight, household response weight, individual selection weight, and individual response weight followed by post-stratification based on census data for age. Weighted averages standardized, and χ^2^ tests were used to compare between adults and children | | | |

**Supplementary** **Figure 1.** Map of municipalities, polygons, participating and nonparticipating households.


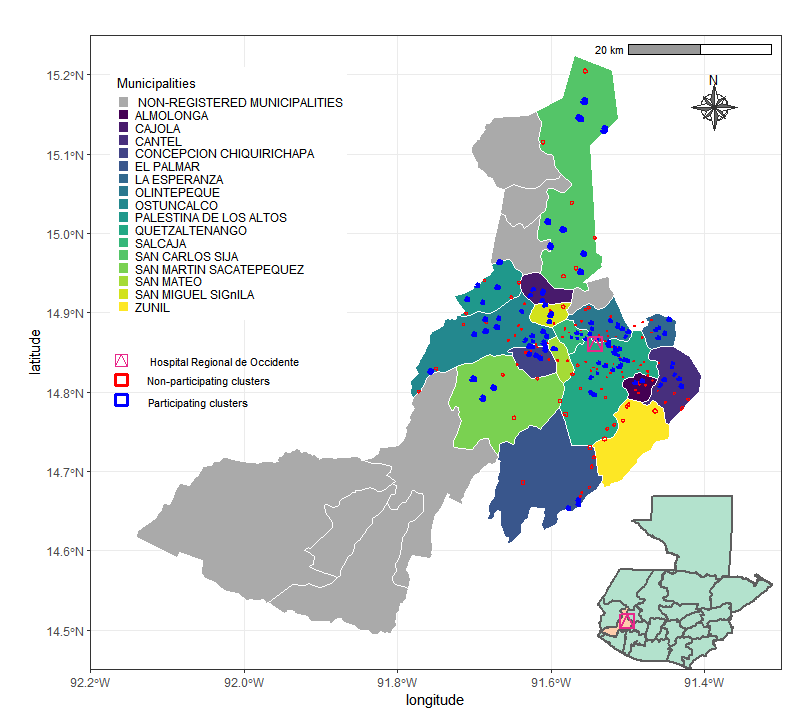


| **Supplementary** **Figure 2.** Enrollment chart for the community and hospital study. |
| --- |
| 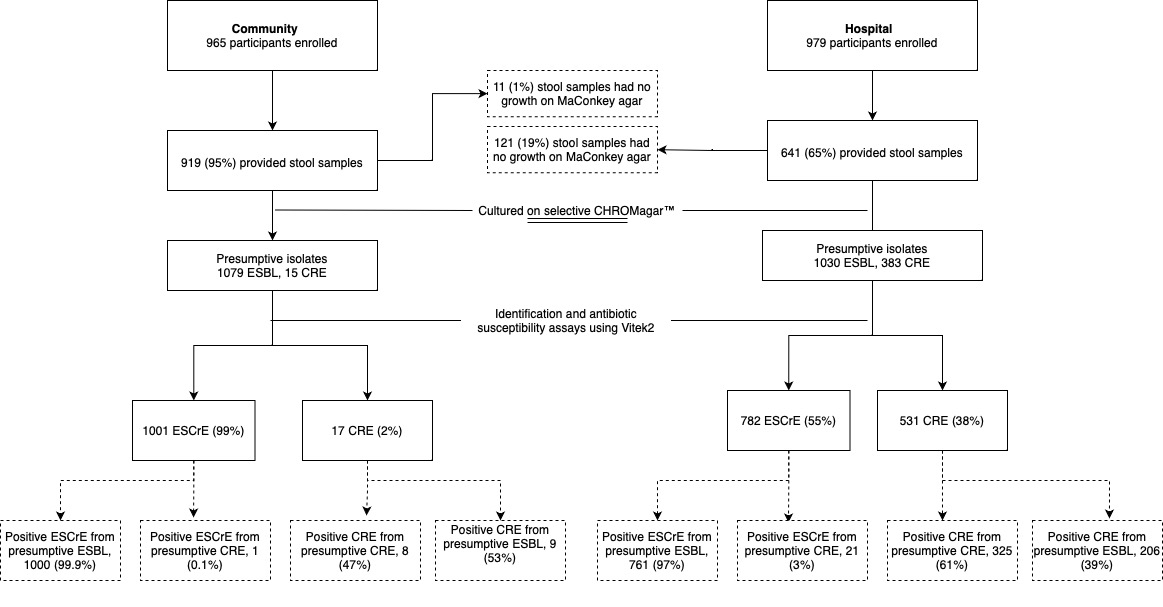 |

| **Supplementary** **Figure 3.** Heatmap showing proportion of hospital CRE isolates that were resistant to 13 antibiotics. This summary was restricted to isolates having unique antimicrobial resistance susceptibility phenotypes on a per participant level to maximize independence between observations. All CRE isolates were non-sensitive or resistant to at least one tested carbapenem (ertapenem, imipenem, or meropenem). Antibiotics shown include amoxicillin with clavulanic acid (Amc), ampicillin (Amp), aztreonam (Atm), cefazolin (Cz), ciprofloxacin (Cip), ceftriaxone (Cro), cefepime (Fep), gentamicin (Gen), levofloxacin (Lev), nitrofurantoin (Nit), sulfamethoxazole & trimethoprim (Sxt), tetracycline (Tet) and tazobactam with piperacillin (Tzp). Ampicillin breakpoints were not available for *Enterobacter* sp. or *Citrobacter* sp. (grey cells). Note that all *Klebsiella* is intrinsically resistant to Amp. Isolates with intermediate resistant were classified as susceptible for this figure. |
| --- |
| 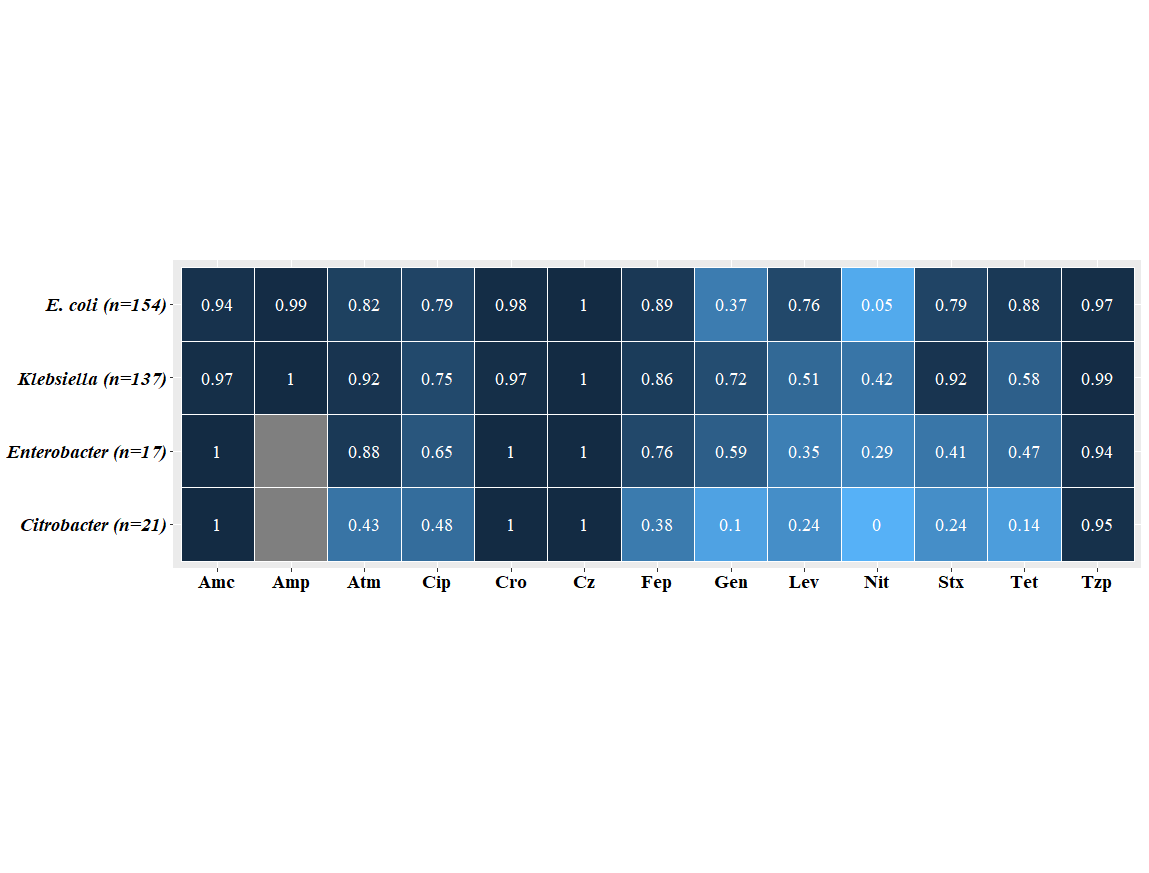 |

| **Supplementary** **Figure 4.** Distribution of antimicrobial resistance phenotypes for CRE isolates collected from hospital participant stool samples. Only one unique antimicrobial resistance susceptibility phenotype was included for each patient-participant combination. Bottom panel indicates the combination of unique resistance phenotypes (black bubbles connected by solid line). The middle histogram provides the frequency of isolates corresponding to each unique combination of resistance phenotypes. The upper panel shows the distribution of resistance phenotypes by proportion of total isolates of that species or genera. Antibiotic abbreviations are found in Table S1. Only combinations of resistance phenotypes for ≥3 isolates are shown for *E. coli* (n=154), *Klebsiella* (n=137), *Enterobacter* (n=17) and *Citrobacter* (n=21). All *Klebsiella* is intrinsically resistant to Amp. |
| --- |
| 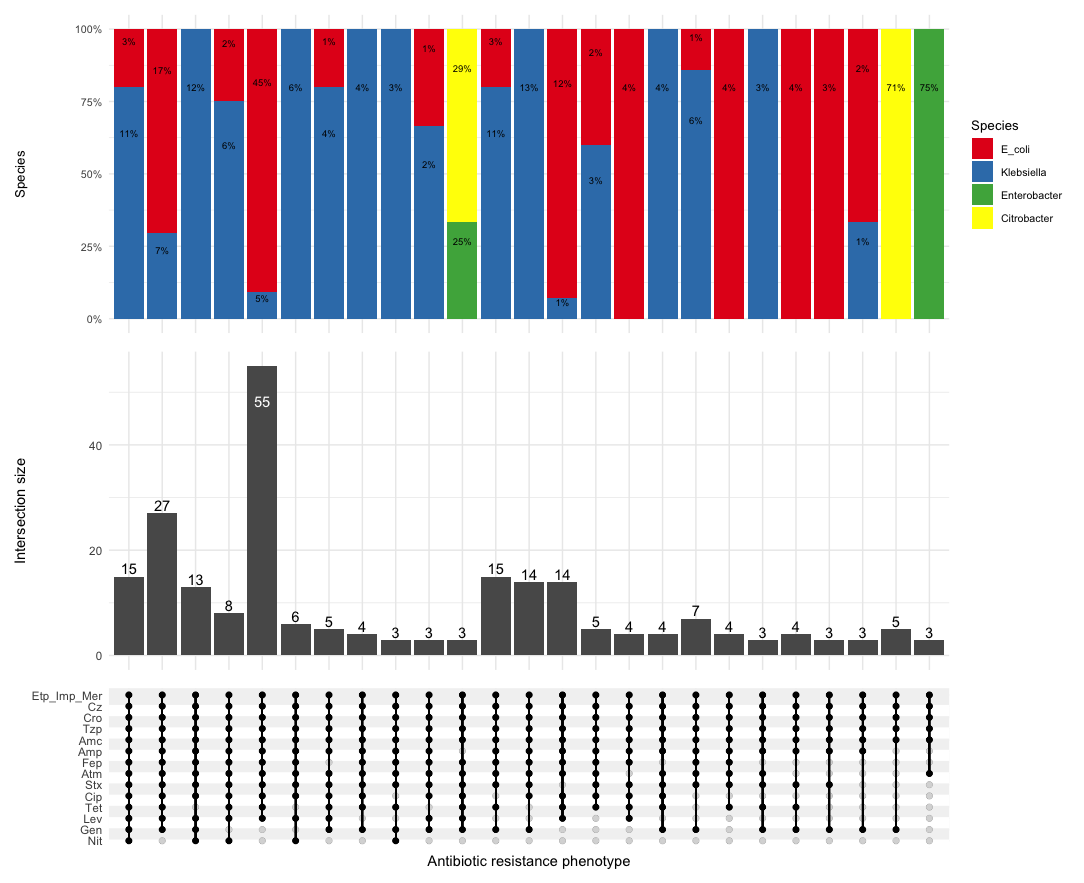 |

| **Supplementary** **Figure 5.** Heatmap showing proportion of hospital and community ESCrE isolates that were resistant to 10 antibiotics. This summary was restricted to isolates having unique antimicrobial resistance susceptibility phenotypes on a per participant level to maximize independence between observations. All ESCrE isolates were resistant to ceftriaxone and sensitive to all tested carbapenems (ertapenem, imipenem, or meropenem) and most ESCrE isolates were also resistant to ampicillin, and cefazolin (not shown). Hospital isolates are designed (“_H”) and community isolates are designated (“_C”). Antibiotics shown include amoxicillin with clavulanic acid (Amc), aztreonam (Atm), ciprofloxacin (Cip), cefepime (Fep), gentamicin (Gen), levofloxacin (Lev), nitrofurantoin (Nit), sulfamethoxazole & trimethoprim (Sxt), tetracycline (Tet) and tazobactam with piperacillin (Tzp). Isolates with intermediate resistant were classified as susceptible for this figure. |
| --- |
| 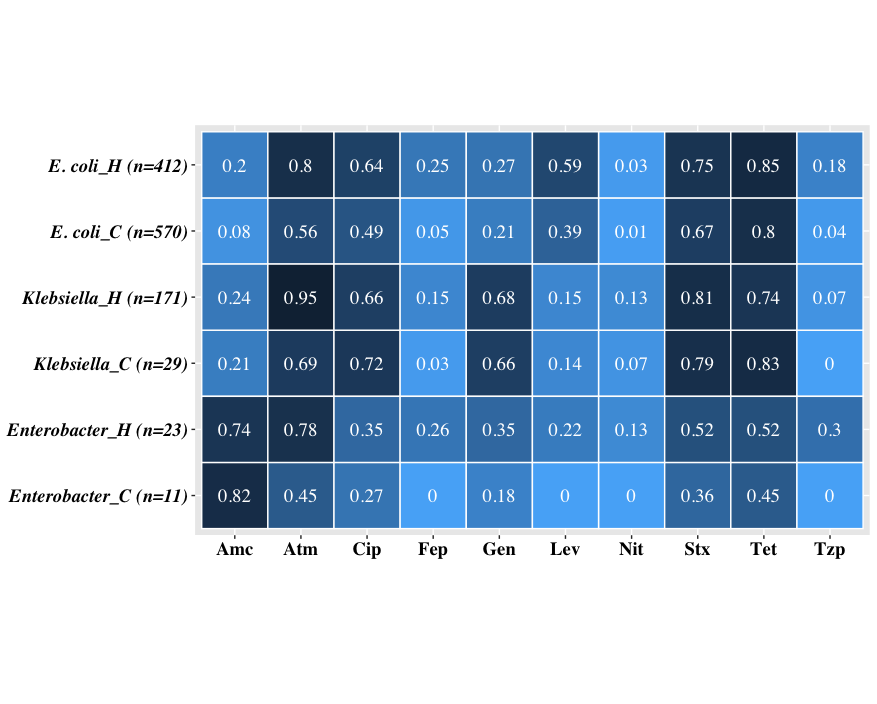 |

| **Supplementary** **Figure 6.** Distribution of antimicrobial resistance phenotypes for ESCrE isolates collected from hospital participant stool samples. Only one unique antimicrobial resistance susceptibility phenotype was included for each patient-participant combination. Bottom panel indicates the combination of unique resistance phenotypes (black bubbles connected by solid line). The middle histogram provides the frequency of isolates corresponding to each unique combination of resistance phenotypes. The upper panel shows the distribution of resistance phenotypes by proportion of total isolates of that species or genera. All isolates were resistant to ampicillin, ceftriaxone and cefazolin (Amp_Cro_Cz). Antibiotic abbreviations are found in Table S1. Only combinations of resistance phenotypes for ≥3 isolates are shown for *E. coli* (n = 412), *Klebsiella* (n = 171), and *Enterobacter* (n = 23). All *Klebsiella* is intrinsically resistant to Amp. |
| --- |
| 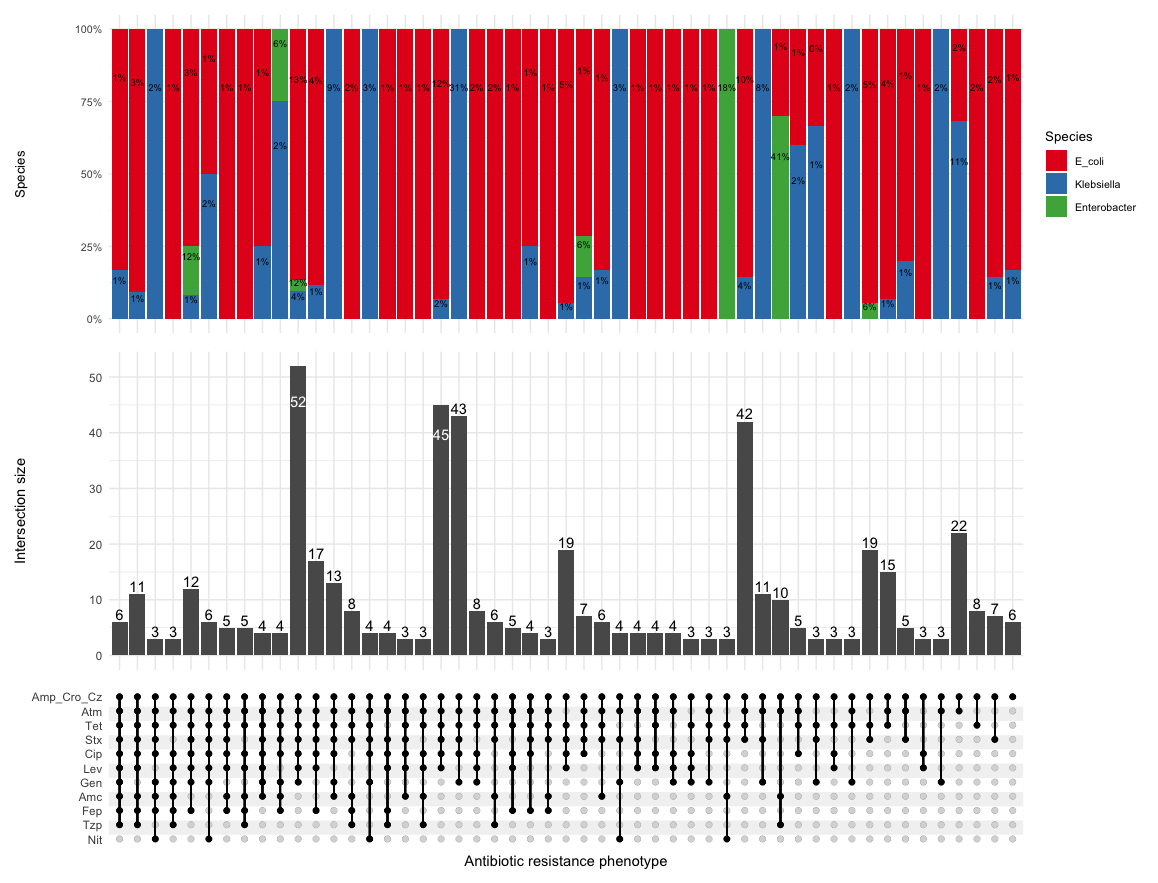 |

| **Supplementary** **Figure 7.** Distribution of antimicrobial resistance phenotypes for ESCrE isolates collected from community participant stool samples. Only one unique antimicrobial resistance susceptibility phenotype was included for each patient-participant combination. Bottom panel indicates the combination of unique resistance types (black bubbles connected by solid line). The middle histogram provides the frequency of isolates corresponding to each unique combination of resistance phenotypes. The upper panel shows the distribution of resistance phenotypes by proportion of total isolates of that species or genera. All isolates were resistant to ampicillin, ceftriaxone and cefazoline (Amp_Cro_Cz). Antibiotic abbreviations are found in Table S1. Only combinations of resistance phenotypes for ≥3 isolates are shown for *E. coli* (n = 570), *Klebsiella* (n = 29), and *Enterobacter* (n = 11). All *Klebsiella* is intrinsically resistant to Amp. |
| --- |
| 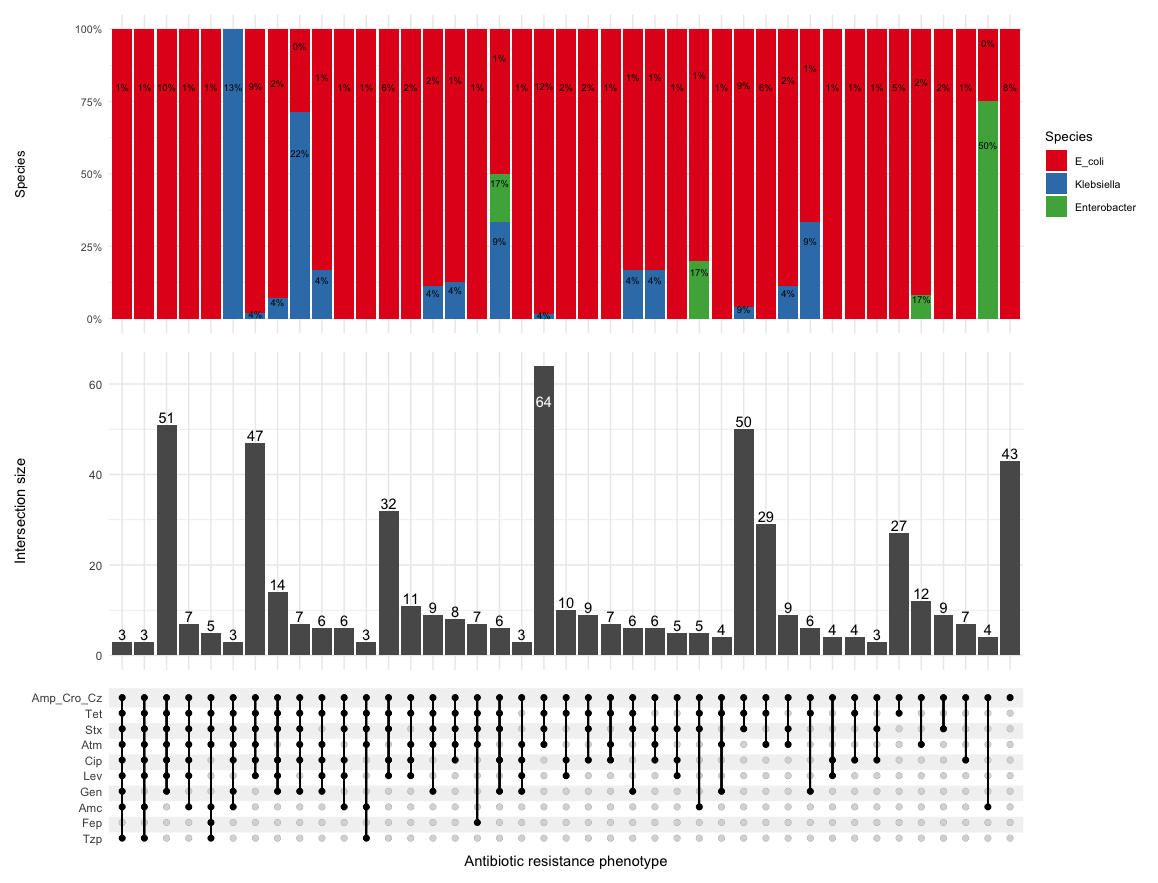 |
